# High-Intensity Interval Training Does Not Change Vaspin and Omentin and Does Not Reduce Visceral Adipose Tissue in Obese Rats

**DOI:** 10.3389/fphys.2021.564862

**Published:** 2021-02-26

**Authors:** Leandro Ribeiro Costa, Cynthia Aparecida de Castro, Diego Adorna Marine, Fernando Fabrizzi, Vanessa de Oliveira Furino, Iran Malavazi, Fernanda de Freitas Anibal, Ana Cláudia Garcia de Oliveira Duarte

**Affiliations:** ^1^Department of Physical Education and Human Motricity – DEFMH, Biological and Health Sciences Center – CCBS, Federal University of São Carlos – UFSCar, São Carlos, Brazil; ^2^Department of Morphology and Pathology – Biological and Health Sciences Center – CCBS, Federal University of São Carlos – UFSCar, São Carlos, Brazil; ^3^Faculty of Philosophy, Sciences and Letters of Penápolis-Brazil, Penápolis, Brazil; ^4^Department of Genetics and Evolution – Biological and Health Sciences Center – CCBS, Federal University of São Carlos – UFSCar, São Carlos, Brazil

**Keywords:** vaspin, **o**mentin, visceral adipose tissue, **h**igh-intensity interval training, **b**ody composition, **o**besity, **h**igh fat diet

## Abstract

This study aimed to determine the expression of omentin and vaspin, inflammatory markers, body composition, and lipid profile in diet-induced obese rats and high-intensity interval training (HIIT). Forty Wistar rats were divided into four groups: untrained normal diet, trained normal diet (T-ND), untrained high-fat diet (Unt-HFD), and trained high-fat diet (T-HFD). For the animals of the Unt-HFD and T-HFD groups, a high-fat diet was offered for 4 weeks. After that, all the animals in the T-ND and T-HFD groups were submitted to HITT, three times per week, for 10 weeks (2 weeks of adaptation and 8 weeks of HIIT). Muscle (gastrocnemius), liver, epididymal adipose tissue, retroperitoneal adipose tissue, visceral adipose tissue (VAT), and serum were collected to analyze TNF-α, IL-6, PCR, IL-8, IL-10, IL-4, vaspin, and omentin. A body composition analysis was performed before adaptation to HIIT protocol and after the last exercise session using dual-energy X-ray absorptiometry. Omentin and vaspin in the VAT were quantified using Western blotting. The results showed that, when fed a high-fat diet, the animals obtained significant gains in body fat and elevated serum concentrations of vaspin and blood triglycerides. The HIIT was able to minimize body fat gain but did not reduce visceral fat despite the increase in maximum exercise capacity. Moreover, there was a reduction in the serum levels of adiponectin, IL-6, and IL-10. Finally, we concluded that, although the training protocol was able to slow down the weight gain of the animals, there was no reduction in visceral fat or an improvement in the inflammatory profile, including no changes in omentin and vaspin.

## Introduction

Obesity is related to a wide range of diseases, such as arterial hypertension, diabetes mellitus type II, some types of cancer, and non-alcoholic hepatic steatosis. These comorbidities can mostly be attributed to metabolic and endocrine alterations occurring in the adipose tissue, from its expansion (Hwang et al., 2015; Shie et al., 2015). Researchers showed that adipocyte hypertrophy results in an abnormal function of the cell, and this remodeling could lead to an alteration in the secretion of metabolites such as adipokines (leptin, adiponectin, vaspin, and omentin), causing adipocyte death, local hypoxia, and influx of fatty acid (Choe et al., 2016).

One of the strategies adopted to mitigate this low-grade chronic inflammation is to reduce body fat, resulting in higher circulating levels of anti-inflammatory cytokines, such as adiponectin, associated with a reduction in pro-inflammatory characteristics. Thus, physical exercise has been shown to be an efficient strategy (Gleeson et al., 2011). Physical exercise has anti-inflammatory characteristics, offering a protective effect against diseases associated with chronic low-grade inflammation present in obesity, reducing the levels of inflammatory cytokines and increasing anti-inflammatory properties (Hermsdorff and Monteiro, 2004; Petersen and Pedersen, 2005; Ghoshal, 2015).

Among the training protocols available, high-intensity interval training (HIIT) has achieved an increase in popularity. HIIT alternates periods of high intensity with active or passive intervals and is a time-efficient strategy suitable to improve cardiorespiratory fitness, reduce cardiometabolic risks, and improve fat oxidation, leading to significant weight loss in obese and overweight populations (Alahmadi et al., 2011; Heydari et al., 2012; Alahmadi, 2014). HIIT generates physiological adaptations including the elevation of mitochondrial content, maximal aerobic capacity, and generation of hypertrophy in skeletal muscle (MacInnis and Gibala, 2017; Robinson et al., 2017). In addition to these benefits, the use of HIIT in obese populations can lead to changes in the inflammatory profile by reducing inflammatory cytokines while increasing the anti-inflammatory properties due to the reduction in body weight and visceral adiposity (Steckling et al., 2015).

This reduction in visceral obesity has been the target of studies since it can cause changes in the expression of important adipokines. Over the last decades, many adipokines have been discovered and are of special interest to researchers for improving the condition of obesity, diabetes, and low-grade inflammation, such as omentin and vaspin. Omentin is adipokine produced by the stromal–vascular fraction of visceral adipose tissue (VAT) and, in low concentrations, by subcutaneous adipose tissue (Yang et al., 2006; De Souza Batista et al., 2007). It has been suggested in the literature that production occurs under glucose and insulin regulation (Komiya et al., 1998) and is modified in several pathological situations, such as obesity and insulin resistance (Kuperman et al., 2005). Due to this fact, a reduction in omentin levels is associated with an increase in metabolic risk factors, suggesting its use as a negative biomarker for obesity (De Souza Batista et al., 2007).

On the other hand, vaspin is a member of the serine protease inhibitor family (Hida et al., 2005; Nakatsuka et al., 2012) and is highly expressed by VAT in obesity conditions as well as subcutaneous adipose tissue (Shaker and Sadik, 2013) and in low quantities by skeletal muscle and liver (Körner et al., 2011; Goktas et al., 2013). However, although the mechanisms of action of vaspin are poorly understood, it is proposed that its action may represent a compensatory mechanism in metabolic abnormalities induced by obesity (Barraco et al., 2014; Proença et al., 2014). Thus, a better understanding of the adjacent mechanisms of exercise in the secretion of adipokines can define more effective strategies to control obesity and co-morbidities.

Therefore, based on the pathophysiological aspects associated with obesity, this study aimed to determine the expression of omentin and vaspin, inflammatory markers, body composition, and lipid profile in diet-induced obese rats and HIIT.

## Materials and Methods

### Ethics and Experimental Groups

The experimental protocol lasted 18 weeks. The experimental procedures in this study conformed to the Committee on Animal Research and Ethics (no. 3963080116) from the Federal University of São Carlos (UFSCar). Adult Wistar male rats (*n* = 40, ≅300 g) were housed in groups (*n* = 4 to 5/cage) with a temperature-controlled environment (22–24°C), humidity of 50–60%, reversed 12/12-h light/dark cycle (lights on at 6 pm), and water and food *ad libitum*. After 4 weeks of acclimatization (90 days), the rats were randomly divided into two groups: normal diet (ND; *n* = 20) and high-fat diet (HFD; *n* = 20), and they were fed for 8 weeks. Then, the animals were randomly distributed into four experimental groups (*n* = 10): untrained normal diet (Unt-ND), trained normal diet (T-CD), untrained high-fat diet (Unt-HFD), and trained high-fat diet (T-HFD).

### Diets

The normal fat diet (in pellet form), containing 4.8% total fat, was used as control diet in the NFD group, as previously reported (Estadella et al., 2004; de Castro et al., 2017, 2019). The palatable high-fat diet was prepared with standard rat chow plus peanuts, milk chocolate, and sweet biscuits in a proportion of 3:2:2:1 (Estadella et al., 2004; de Castro et al., 2017, 2019). All components were powdered and mixed to form pellets. This diet is composed of 20% fat (Table 1) and was standardized by Estadella et al. (2004); since then, it has been used to induce obesity phenotype in Wistar rats (de Castro et al., 2017, 2019). The nutritional composition of the diet was analyzed by CBO Laboratories of Analyzes Ltda., Valinhos-SP, Brazil. It is worth mentioning that the use of the term high-fat diet is due to the increase in fat due to the standard diet that has 4% fat *vs* 20% (HFD), being efficient in the study of obesity (Bruder-Nascimento et al., 2013; Moreno-Fernández et al., 2018; Li et al., 2020).

**TABLE 1:** Nutritional composition of the diet.

	**HFD**	**ND**
Energy value (cal/g)	4,665.00	3,854.00
Moisture and volatiles (%)	14.72	12.47
Fat (%)	20	4.80
Carbohydrates (%)	32.90	39.23
Proteins (%)	18.12	22.81
Fibers (%)	2.97	5.82
Minerals (%)	3.29	6.87
Potassium (%)	0.60	1.26
Calcium (%)	0.52	1.2^*a*^
Sodium (%)	0.14	0.22
Vitamin A (UI/kg)	1,149.00	25,000.00^*a*^
Vitamin D3 (UI/kg)	836.90	4,000.00^*a*^
Vitamin E (mg/kg)	229.94	80.00^*a*^
Maltose (%)	Undetectable	Undetectable
Free xylose (%)	Undetectable	Undetectable
Free glucose (%)	0.12	Undetectable
Free fructose (%)	0.17	Undetectable
Sucrose (%)	12.49	1.65
Lactose (%)	1.88	Undetectable
Free galactose (%)	Undetectable	Undetectable
Raffinose (%)	0.30	0.74

*ND, normal diet; HFD, high-fat diet. The undetectable values were below the threshold of quantification (<100 mg/kg, 0.1%). ^*a*^Nutritional information obtained from the manufacturer.*

### Body Mass and Food Intake Measurement

The body mass (BM) was measured once a week, and food intake was measured every 2 to 3 days, between 8 and 12 h. Diet intake was calculated by the difference in weight between the amount of food offered and the amount of food remaining.

### HIIT Protocol

#### Adaptation

The animals were adapted to a treadmill for 2 weeks. The animals of the training group ran on the treadmill between 10 and 20 m/min. In order to simulate a similar environment to training, the untrained animals were also placed on the treadmill so that they could adapt.

#### Maximum Exercise Capacity

After the adaptation, a maximum exercise capacity (MEC) test was performed. The animals started to run on the treadmill at 6 m/min with 25% incline for 5 min, with an increase of 0.5 m/min every 2 min until the maximum speed was obtained. As a criterion for determining exhaustion, the interruption was the moment when the animal was no longer able to run by increasing the speed of the treadmill (Wisløff et al., 2001; Høydal et al., 2007).

#### HIIT Protocol

The HIIT protocol consisted of three exercise sessions per week for 8 weeks. The training was preceded by a 5-min warm-up, with the animals running at 40% of the MEC and then the alternation between high intensity for 4 min (85–95% of MEC) and recovery for 3 min (40–50% of MEC), with a maximum of six intermittent intervals. Every 2 weeks, another incremental test to determine the MEC was carried out again to adjust the intensities of the exercise (Haram et al., 2009; Kemi et al., 2015; Songstad et al., 2015). Throughout the procedure, electric shocks were not used as a form of stimulation.

### Experiment and Sample Collection

The animals were euthanized by decapitation using a guillotine after 8 h of fasting. The trained animals were sacrificed 48 h after the last exercise session. VAT, epididymal (EPI) adipose tissue, retroperitoneal (RET) adipose tissue, brown adipose tissue (BAT), liver, gastrocnemius muscle, and serum were collected, dissected, weighed, and stored in a freezer at −80°C for posterior biochemical and morphometric analyses.

### Dual-Emission X-Ray Absorptiometry

Body composition evaluation was performed before adaptation to the HIIT protocol and after the last exercise session. The animals were anesthetized with ketamine (40 mg/kg) and xylazine (5 mg/kg; IACUC) and were later placed in prone position for them to be scanned using the dual-energy X-ray absorptiometry (DXA)–dual-range emission densitometry (Lunar iDXA 200368 GE^®^ instrument, Lunar, WI, United States). BM, body fat, fat mass, and fat-free mass values were obtained. Image analyses were performed using the Encore 2008, 12.20 GE, HEALTHCARE.

### Western Blotting to Determine Omentin and Vaspin

Omentin and vaspin in the VAT were quantified using Western blotting. The tissues were processed to obtain the total protein extract using an extraction buffer [sodium dodecyl sulfate (SDS), 0.1% (p/v); Triton, 1% (v/v); Tris–HCl, pH 7.5, 50 mM; NaCl, 150 mM; EDTA, 15 mM; EGTA, 5 mM; NaF, 100 mM; and Na_2_P_2_O_7_, 10 mM] as well as protease inhibitors (Complete-Mini Roche^®^ 1×). The concentration of protein was quantified using Lowry’s colorimetric method (1951). The crude protein extracts for each experiment were submitted to SDS–polyacrylamide gel electrophoresis (12%) and Tris-glycine buffer 1 × (Laemmli’s method) using a vertical electrophoresis tank (BioRad). The proteins were then transferred from the gel to the nitrocellulose membrane (0.45 μm, BioRad) in a submerged transfer procedure according to the manufacturer’s protocol. Membrane blockage was done with Tris-buffered saline with 0.1% Tween^®^ 20 (TBST) 1 × containing 9% of milk powder for 4 h at room temperature. The membranes were then incubated, overnight at 4°C, with the primary antibody anti-omentin (1-1000, sc-104334, and Santa Cruz^®^) and anti-vaspin (1-1000, sc-79815, and Santa Cruz^®^) TBST 1 × containing 5% of milk powder. The membrane was incubated with a secondary anti-goat IgG-HRP antibody: (1-3000, sc-2020) in TBST 1×, immunodetection was performed using a chemiluminescence kit (ECL Prime, GE Healthcare^®^, Life Sciences). The blot image was acquired using the Chemidoc (BioRad^®^) equipment. Protein concentrations were normalized by using GAPDH diluted 1:10,000 (Abcam^®^) in VAT. All the membranes were normalized using an intra-membrane control.

### Quantifications of Cytokines and Adipokines

The quantifications of omentin, vaspin, TNF-α, IL-6, IL-8, IL-10, C-reactive protein, and adiponectin were performed from serum and determined by enzyme-linked immunosorbent assay (ELISA) method following the specifications corresponding to the kits. For the cytokine analyses, such as IL-4, IL-10, IL-6, and TNF-α, OptEIA (BD Biosciences^®^) kits were used; for the IL-8, PCR and adiponectin analyses, DuoSet ELISA kits were used (R&D Systems^®^); for the omentin and vaspin analyses, EIA-OME and EIA-VAP (RayBiotech^®^) kits were used. The concentrations of the samples were calculated from the titration curve of the cytokine patterns, and the final concentrations were expressed in pg/ml or ng/ml depending on the kit.

### Statistical Analysis

All statistical analyses were performed using the Sigma Stat Software (version 3.5). Data normality was verified by the Kolmogorov–Smirnov test; equality of variance (Levene’s method) and non-parametric tests were used when the data did not present normal distribution and/or equality of variance. Comparisons among the groups were made using two-way ANOVA. Tukey multiple-comparison test was used when the two-way ANOVA test detected a statistical difference. Independent *t*-test was used for comparisons between two independent groups. The level of significance was set at 5% (*p* < 0.05).

## Results

### Body Mass and Food Consumption

The animals fed on a high-fat diet presented significantly higher BM than their respective controls at the end of the experiment. The Unt-HFD and T-HFD groups presented lower caloric intake when compared to the Unt-ND and T-ND groups, respectively, (Table 2). In addition, the Unt-HFD group showed significantly higher triglyceride values when compared to the Unt-ND group.

**TABLE 2:** Body mass gain and food intake.

**Groups**	**Body mass (g)**			**Caloric intake (kcal/day)**	**Triglycerides (mg/dl)**
	**Initial**	**Final**	**Body mass gain**		
Unt-ND	280.90 ± 15.50	527.80 ± 43.88^d^	246.90 ± 36.85	121.98 ± 2.84	34.51 ± 10.04
T-ND	273.80 ± 23.69	497.40 ± 31.74^d^	223.6 ± 23.32	118.44 ± 2.67	30.96 ± 8.09
Unt-HFD	277.40 ± 19.54	619.00 ± 77.94^ad^	341.90 ± 72.42^a^	114.89 ± 3.59^a^	50.88 ± 11.73^a^
T-HFD	282.50 ± 14.57	584.70 ± 49.51^bd^	302.20 ± 56.18^b^	109.87 ± 3.34^b^	42.08 ± 9.09

*Unt-ND, untrained normal diet (*n* = 10); T-ND, trained normal diet (*n* = 10); Unt-HFD, untrained high-fat diet (*n* = 10); and T-HFD, trained high-fat diet (*n* = 10). Data presented as mean ± SD (*p* < 0.05). ^a^*vs*. Unt-ND. ^b^*vs*. T-ND. ^d^Initial *vs*. final.*

### Maximum Capacity of Exercise

The training variables are presented in Table 3. The MEC at the beginning of the training protocol was significantly different between the T-ND and T-HFD groups, 9.1% lower in the T-HFD group. At the end of 10 weeks, this training capacity was higher in relation to the first MEC test, showing an improvement of this variable for the animals trained with HIIT, but there was no difference between the T-ND and T-HFD groups in the post-exercise condition (Table 3). The same behavior occurred with the variable time to exhaustion, but the distance covered was greater in T-ND compared to T-HFD group. Interestingly, the MEC in the T-ND group was 228%, and in the T-HFD group it was 235%; there was no difference in the post-exercise. Therefore, it showed 16% improvement compared to T-ND.

**TABLE 3:** Variables of exercise.

**Variables**		**T-ND**	**T-HFD**
Pre-exercise	MEC (m/min)	13.74 ± 0.87	12.5 ± 0.95^a^
	Time to exhaustion (min)	28.66 ± 1.65	25.77 ± 3.15^a^
	Distance covered (m)	393.78 ± 1.43	322.12 ± 2.99^a^
Post-exercise	MEC (m/min)	31.36 ± 3.63^*b*^	29.25 ± 2.67^b^
	Time to exhaustion (min)	27.00 ± 6.60	20.20 ± 10.15^b^
	Distance covered (m)	846.70 ± 24.00^b^	590.85 ± 27.10^ab^
Δ of MEC (%)		228	235

*Unt-ND, untrained normal diet (*n* = 10); T-ND, trained normal diet (*n* = 10); Unt-HFD, untrained high-fat diet (*n* = 10); and T-HFD, trained high-fat diet (*n* = 10). Data presented as mean ± SD (*p* < 0.05). ^a^*vs.* T-ND. ^b^Pre-exercise *vs.* post-exercise in the same group.*

### Body Composition

**FIGURE 1:**
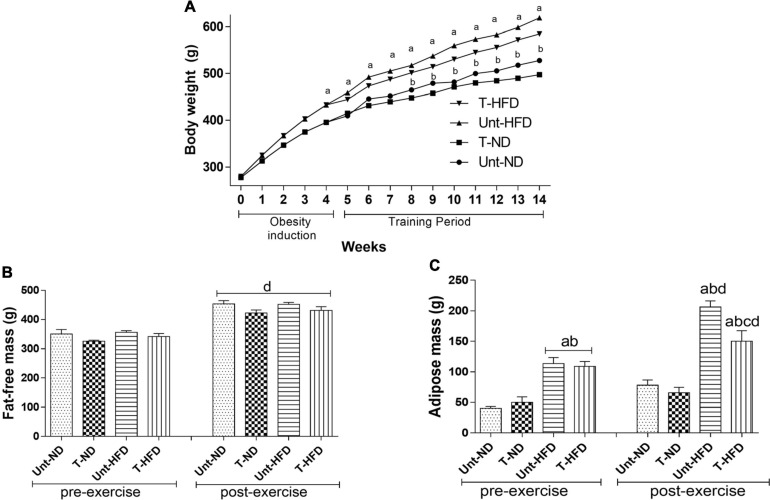
Body composition. **(A)** The evolution of body weight gain, **(B)** fat-free mass pre- and post-exercise, and **(C)** adipose mass pre- and post exercise. Unt-ND, untrained normal diet (*n* = 10); T-ND, trained normal diet (*n* = 10); Unt-HFD, untrained high-fat diet (*n* = 10); and T-HFD, trained high-fat diet (*n* = 10). Data presented as mean ± SD (*p* < 0.05). ^*a*^*vs.* Unt-ND; ^*b*^*vs.* T-ND; ^*c*^*vs.* Unt-HFD; and ^*d*^pre-exercise *vs.* post-exercise.

Figure 1A shows that, from the fourth week, the Unt-HFD group weight was significantly higher than the untrained normal diet group (Unt-ND). After the experimental protocol, it was found that training was not able to promote significant changes in the T-HFD group. However, it presents 5.72% lower value for this variable than the Unt-HFD group. The animals’ fat-free mass, assessed by DXA, was higher in all groups after 10 training weeks, but there was no significant change between groups for this parameter considering only the end of training (Figure 1B).

Regarding adipose mass (Figure 1C), it was observed that, at the end of the diet-induced obesity (pre-exercise), the Unt-HFD and T-HFD groups showed higher values when compared to the respective control groups (Unt-ND and T-ND). After 10 weeks of training, a similar pattern was observed as the groups that were fed a high-fat diet (Unt-HFD and T-HFD) showed higher values of body fat compared to the Unt-ND and T-ND groups. However, the T-HFD group showed significantly lower body fat values compared to the Unt-HFD.

### Relative Weights of Depots

The relative weights of visceral, RET, and EPI abdominal fat depots and BAT were higher in the groups fed a high-fat diet when compared to groups fed a normal diet. The exercise, as observed in Table 4, was not able to cause changes in these fat depots. The Unt-HFD and T-HFD groups showed lower relative weights of the hepatic and muscular tissues when compared to the Unt-ND and T-ND groups, without significant changes between the HFD groups. The diet led to a reduction in the relative weight of the hepatic and muscle tissues since the Unt-HFD group had lower values than the Unt-ND and T-ND, and those of the T-HFD group were less than those of the T-ND. For these tissues, the proposed exercise model was also not able to cause significant changes.

**TABLE 4:** Relative weight of abdominal fat depots and organs (g/100 g body weight).

**Groups**	**Abdominal fat depots**			**BAT**	**Liver**	**Muscle**
	**EPI**	**RET**	**VAT**			
Unt-ND	1.39 ± 0.10	1.35 ± 0.15	0.91 ± 0.08	0.04 ± 0.005	2.71 ± 0.19	0.47 ± 0.03
T-ND	1.25 ± 0.14	1.23 ± 0.21	0.68 ± 0.06	0.05 ± 0.005	2,78 ± 0.18	0.48 ± 0.03
Unt-HFD	2.70 ± 0.14^a^	2.99 ± 0.23^a^	1.81 ± 0.17^a^	0.08 ± 0.006^a^	2.32 ± 0.33^a^	0.40 ± 0.05^a^
T-HFD	2.79 ± 0.14^b^	3.22 ± 0.20^b^	1.65 ± 0.10^b^	0.08 ± 0.008^b^	2.38 ± 0.17^b^	0.42 ± 0.04^b^

*Unt-ND, untrained normal diet (*n* = 10); T-ND, trained normal diet (*n* = 10); Unt-HFD, untrained high-fat diet (*n* = 10); T-HFD, trained high-fat diet (*n* = 10); EPI, epididymal adipose tissue; RET, retroperitoneal adipose tissue; VAT, visceral adipose tissue; and BAT, brown adipose tissue. Data presented as mean ± SD (*p* < 0.05). ^a^*vs*. Unt-ND. ^b^*vs*. T-ND.*

### Cytokines

**FIGURE 2:**
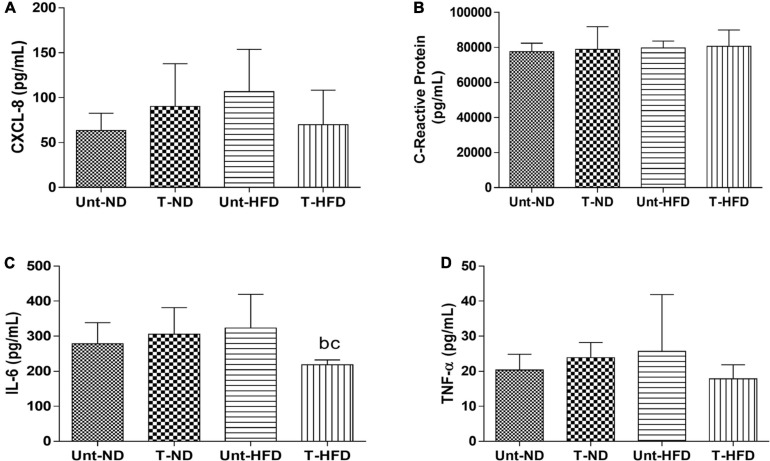
Serum concentrations of pro-inflammatory cytokines. **(A)** CXCL-8: interleukin 8, **(B)** PCR: C-reactive protein, **(C)** IL-6: interleukin 6, and **(D)** TNF-α: tumor necrosis factor alpha. Unt-ND, untrained normal diet (*n* = 10); T-ND, trained normal diet (*n* = 10); Unt-HFD, untrained high-fat diet (*n* = 10); and T-HFD, trained high-fat diet (*n* = 10). Data presented as mean ± SD (*p* < 0.05). ^*b*^*vs.* T-ND; and ^*c*^*vs.* Unt-HFD.

**FIGURE 3:**
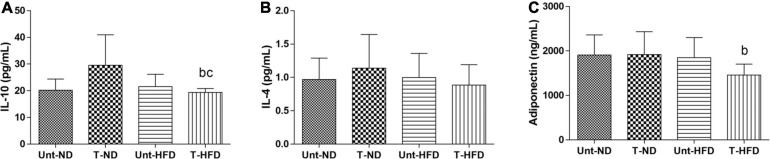
Serum concentrations of anti-inflammatory cytokines. **(A)** IL-10: interlecucin-10, **(B)** IL-4: interleukin-4, and **(C)** adiponectin. Unt-ND, untrained normal diet (*n* = 10); T-ND, trained normal diet (*n* = 10); Unt-HFD, untrained high-fat diet (*n* = 10); and T-HFD, trained high-fat diet (*n* = 10). Data presented as mean ± SD (*p* < 0.05). ^*b*^*vs.* T-ND; and ^*c*^*vs.* Unt-HFD.

In Figure 2C, it can be observed that serum IL-6 was reduced in the T-HFD group when compared to the Unt-HFD and T-ND groups. Besides that, there were no significant changes due to exercise and diet in the values for cytokines CXCL-8, PCR, and TNF-α (Figures 2A,B,D). It is shown that adiponectin decreased in the trained T-HFD compared to the Unt-ND and T-ND (Figure 3C). Besides that, serum IL-10 is reduced in the T-HFD compared to the T-ND group and Unt-HFD group (Figure 3A). Serum IL-4 showed no statistical differences between the groups (Figure 3B).

### Vaspin and Omentin

**FIGURE 4:**
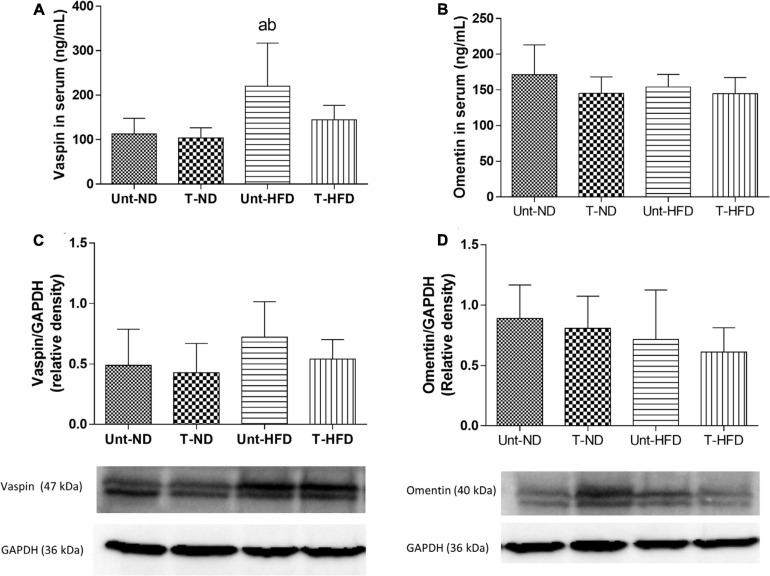
Behavior of serum adipokines and adipose tissue. **(A)** Serum vaspin concentration, **(B)** serum omentin concentration, **(C)** Western blot representative of vaspin (47 kDa) in visceral adipose tissue (VAT), and **(D)** Western blot representative of omentin (40 kDa) in VAT. Unt-ND, untrained normal diet (*n* = 10); T-ND, trained normal diet (*n* = 10); Unt-HFD, untrained high-fat diet (*n* = 10); and T-HFD, trained high-fat diet (*n* = 10). Data presented as mean ± SD (*p* < 0.05). ^*a*^*vs.* Unt-ND; ^*b*^*vs.* T-ND.

The vaspin serum concentration increased upon obesity induction, but it was not changed after the HIIT protocol (Figure 4A). In VAT, the values of vaspin were not altered either by the diet or by the exercise (Figure 4C). Considering the response of omentin in the experimental groups, no statistical differences were found in both serum (Figure 4B) and VAT (Figure 4D).

## Discussion

This study reports the response to omentin and vaspin of Wistar rats fed or not with a high-fat diet and under HIIT. We expected that HIIT could cause metabolic adaptations in adipose tissue, promoting changes in the concentration of omentin and vaspin in obese animals. However, we do not confirm these results. Although the training protocol was able to slow down the weight gain of the animals, there was no reduction in visceral fat or an improvement in the inflammatory profile.

In the present study, the high-fat diet induced an increase in body weight, serum triglycerides, all visceral depots and organs evaluated, and fat mass. We also highlight that, although there were no differences between most cytokines, there was an increase in vaspin in obese animals, which has been used as a biomarker of adiposity, and a reduction of adiponectin in HFD animals. These data together show the effective induction of obesity, supporting the studies already described in the literature (Estadella et al., 2004; Duarte et al., 2008; Sene-Fiorese et al., 2008; Speretta et al., 2012, 2016; Suk and Shin, 2016; Diogo et al., 2020). In addition, the diet promoted a decrease in the food intake of these animals. This behavior was observed in previous investigations and is justified by the increased caloric density of the high-fat diet, which results in a greater satiety of the animals when compared with the commercial chow diet (Estadella et al., 2004; Zambon et al., 2009; Rocha et al., 2016).

Given the small participation of HIIT in slowing down the gain of adipose mass, BM, and triglyceridemia of animals fed a high-fat diet, we believe that these adjustments may be related not only to exercise (Sene-Fiorese et al., 2008; Speretta et al., 2012; Ramos-Filho et al., 2015; MacInnis and Gibala, 2017) but also to the increase in the supply of fats provided by the type of diet. We consider that the energy expended by the animals during the exercise equalized the excessive energy consumption offered in the high-fat diet, thus avoiding a greater accumulation of BM.

Our data reinforced the lipogenic activity generally observed from the consumption of high-fat diet since the animals had an increased rate of lipid anabolism, resulting in the high accumulation of fat in the depots EPI, RET, and VAT (Estadella et al., 2004; Sene-Fiorese et al., 2008; Speretta et al., 2012). Besides the increase in abdominal adipose tissue, the diet caused an increase in the BAT, which was already expected since diets rich in fat are able to elevate the thermogenic activity of BAT, leading to a greater synthesis of UCP1. As a consequence, there is an increase in the weight of this tissue (LeBlanc and Labrie, 1997; Estadella et al., 2004). In addition, an increase in liver lipids was observed, probably due to an increase in lipogenesis, or a decrease in beta oxidation (Gauthier et al., 2015). Surprisingly, although the high-fat diet effectively promoted obesity, the liver weight was reduced, which was not commonly observed by other studies using the same type of diet (Duarte et al., 2008; de Castro et al., 2017). This finding can be partially explained by the increase in hepatic triglyceride concentrations (Sene-Fiorese et al., 2008). This accumulation displaces the predominance of fatty acids as the main energy substrate in organic reactions to the detriment of hepatic glycogen. In turn, the reduction of glycogen levels in the liver, as observed in this study, may be associated with lower liver weight since glycogen carries water molecules for its transport, and this would significantly increase the weight of the liver (Zambon et al., 2009; Gauthier et al., 2015).

It is important to mention that exercise capacity and adaptations have also been measured indirectly through distance covered and time to exhaustion (Kemi et al., 2015). Both animals that were fed a standard diet (228%) and a high-fat diet (235%) achieved increases in MEC, showing evidence of the adjustments promoted by the proposed exercise model as previously described (Kemi et al., 2015). However, the effects of HIIT on adipose depots (EPI, RET, VAT, and BAT), liver, and muscle proved to be inefficient since the weights of these tissues were not changed by HIIT but only by diet. These results suggest a possible mobilization of free fatty acids from other depots of white adipose tissue during exercise, such as the subcutaneous tissue (Maillard et al., 2019; Motta et al., 2019).

As the exercise did not cause changes in the abdominal fat depots, we already expected that HIIT would also not change the concentration of omentin and vaspin in VAT and circulation. However, an interesting fact was that HIIT was able to reduce the serum levels of adiponectin, IL-10, and IL-6. This dynamic, which was presented by the anti-inflammatory molecules because of the physical exercise used, seems to be related to the mobilization of different fat depots as well as different tissues that secrete these cytokines such as the muscle itself (Pedersen and Febbraio, 2012).

The secretion of inflammatory cytokines is altered in obesity as a compensatory way to mitigate the deleterious effects resulting from obesity (You and Nicklas, 2008; Stefanyk and Dyck, 2010; Golbidi and Laher, 2014). Concerning omentin and vaspin, it was observed in the present study that the training was not able to promote changes in its production in all groups, regardless of the type of diet. These data have been observed previously in our research group (de Castro et al., 2019). The positive values of omentin in their work were observed only in diabetic animals (type 2) submitted to aerobic exercise when compared to combined and resistance exercise. Because it does not mobilize visceral fat, it can be hypothesized that the exercise model proposed in our study was not able to bring about changes in omentin and vaspin within the proposed time. Thus, we assume that the mobilization of these adipokines may be more related to visceral adipose depots.

To confirm the predominance of secretion by adipose tissue, we evaluated the serum and protein expression of adipokines in VAT. We observed that vaspin, but not omentin, was responsive to the induction of obesity, which increases its serum concentration considering the expansion of fat deposits, as previously reported (Shaker and Sadik, 2013; Dimova and Tankova, 2015). Adipocytokines such as omentin and vaspin may be involved with inflammation and have different expressions in eutrophic and obese individuals (Hida et al., 2005; de Castro et al., 2019). These adipokines have been studied as pathological biomarkers because they relate to insulin resistance (Flehmig et al., 2014). Thus, we monitored adipose tissue expansion by the values of serum vaspin in high-fat diet animals. However, despite finding an increase in the expression of vaspin mRNA in obese women compared to eutrophic women, no positive correlation with obesity was observed (Auguet et al., 2011). In general, studies with vaspin still show controversial results as some point to the lack of a relationship between the serum levels and obesity and the distribution of fat, while others demonstrated a positive correlation (Blüher, 2012). Moreover, the hypothesis that vaspin may be directly related to the consumption of excess lipids is not ruled out, thus triggering possible insulin resistance, arteriosclerosis, and heart problems (Derosa et al., 2013; Szkudelska et al., 2014). The increase in serum vaspin without changing its expression in VAT may be due to a greater contribution from the subcutaneous adipose tissue, as previously shown (Jung et al., 2011; Shaker and Sadik, 2013; Weiner et al., 2019).

Regarding omentin, these results were not expected since earlier data showed evidence that omentin can be significantly reduced in obese rats when compared to non-obese rats (De Souza Batista et al., 2007; Feng et al., 2013; Proença et al., 2014). There is no consensus in the literature regarding the concentration of omentin in patients with obesity because their response has not yet been fully elucidated (Derosa et al., 2013; Escoté et al., 2017; Aliasghari et al., 2018). Just as HIIT slowed down fat gain, but this loss was not seen in abdominal fat depots, we suggest that this exercise may have mobilized more subcutaneous fat, which did not reflect the change in adipokines omentin and vaspin.

The importance of these adipokines lies in the fact that they can influence adipocytes and other tissues in an autocrine or paracrine manner, affecting multiple metabolic processes such as regulating eating behavior, insulin sensitivity, inflammation, and immunity (Escoté et al., 2017). Thus, obesity is directly associated with low-grade chronic inflammation, as the expression of proinflammatory cytokines (IL-6, IL-8, PCR, and TNF-α) is shown to be increased in this pathology. Studies show that there is a close link between cytokines from obesity and the development of other chronic diseases (de Leal and Mafra, 2013; Mraz and Haluzik, 2014). However, in the present study, no inflammatory condition was observed in animals that were fed the high-fat diet. It is believed that this situation was due to the short time of exposure of the animals to the high-fat diet and that the accumulation of fat was not sufficient to cause changes in the production of such adipokines with pro-inflammatory characteristics (Rocha et al., 2016). This was contrary to other studies found in the literature (Tzanavari et al., 2010; Jung and Choi, 2014).

Even with this slight inflammatory activation caused by the diet, it is noteworthy that HIIT in obese animals reduced the levels of IL-6, IL-10, and adiponectin. It is known that the stress promoted by physical exercise is linked to an increase in catecholamine discharge and that the catecholamine receptors present in macrophages have great importance in modulating the inflammatory response (Figueiredo et al., 2017). In the condition of obesity, a high-fat diet could lead to an increase in catecholamines, which, in turn, *via* cAMP response element-binding protein, would suppress the expression of adiponectin (Liu and Liu, 2009). Thus, it can be considered that the lower values of adiponectin and IL-10 in trained animals may also mean that the training was intense for the metabolic condition of obese animals.

The effects of HIIT in the systemic inflammatory profile are controversial since serum levels may arise not only from adipose tissue but also from muscle, liver, and others. Studies show that this reduction in adiponectin in obese individuals may contribute to the susceptibility to viral lung infections and the severity of these infections in obese individuals (Salvator et al., 2020). Despite the benefits of adipokine in protecting against metabolic diseases such as obesity and diabetes (Jortay et al., 2012; Martinez-Huenchullan et al., 2020), it is important to note that there are different adiponectin isoforms with functions that are not entirely clear, and exercise seems to regulate each isoform differently (Gerosa-Neto et al., 2016; Martinez-Huenchullan et al., 2018). Further studies are needed to verify the role of different exercise modalities in circulating adipokines and cytokines.

The potential limitations include failure to assess the thermogenic effects of HIIT on whole-body fat metabolism (often done by direct or indirect calorimetry or oxygen uptake and carbon dioxide gas exchange measurement). However, exercise performance improvement in both HFD and NFD animals suggests the negligible thermogenic effects of HIIT on adipose tissues. Still HIIT fat loss thermogenic effects and adipose tissue mobilization need further investigation. Another potential limitation is that, although in this study we used a lower fat (20%) content than other studies to induce obesity, previous results showed that our diet was effective to promote obese phenotype, including augmentation in body adiposity, body weight, weight gain, total mass, and visceral depots (Estadella et al., 2004; Duarte et al., 2008; Sene-Fiorese et al., 2008; Oishi et al., 2018).

In summary, we suggest that vaspin and omentin are not responsive to HIIT in obese and eutrophic animals, although the training protocol was able to retard the weight gain, with no change in visceral abdominal fat and no improvement in the inflammatory profile. Further studies are needed to explore the molecular mechanisms involved in the expression of omentin and vaspin in response to exercise.

## Data Availability Statement

The raw data supporting the conclusions of this article will be made available by the authors, without undue reservation.

## Ethics Statement

The experimental procedures in this study conformed to the Committee on Animal Research and Ethics (n°. 3963080116) from the Federal University of São Carlos (UFSCar).

## Author Contributions

LC, AD, CC, and FF helped conceive the design, performed the analyses, analyzed the data, and wrote the first draft of the manuscript. IM and FA performed other data analyses and helped to draft the manuscript. DM, VF, LC, and CC helped conceive the design and supervised the experimental trials and training sessions. LC, DM, VF, CC, FA, and FF helped with data analyses and helped draft the manuscript. DM, CC, FF, and AD interpreted the study results and edited the manuscript. LC, DM, VF, CC, FF, and AD helped conceive the design, helped with the data analyses, provided funding for the study, and helped draft the manuscript. All the authors have read and approved the final version of the manuscript and agreed with the order of presentation of the authors.

## Conflict of Interest

The authors declare that the research was conducted in the absence of any commercial or financial relationships that could be construed as a potential conflict of interest.
